# Vascular homeostasis in atherosclerosis: A holistic overview

**DOI:** 10.3389/fimmu.2022.976722

**Published:** 2022-09-12

**Authors:** Suowen Xu, Qing Rex Lyu, Iqra Ilyas, Xiao-Yu Tian, Jianping Weng

**Affiliations:** ^1^ Department of Endocrinology, Institute of Endocrine and Metabolic Diseases, The First Affiliated Hospital of USTC, Division of Life Sciences and Medicine, Clinical Research Hospital of Chinese Academy of Sciences (Hefei), University of Science and Technology of China (USTC), Hefei, China; ^2^ Medical Research Institute, Chongqing General Hospital, Chongqing, China; ^3^ School of Biomedical Sciences, Chinese University of Hong Kong, NT, Hong Kong SAR, China

**Keywords:** aorta zonation, atherosclerosis, homeostasis, vascular ecosystem, endothelial function

## Abstract

Atherosclerosis refers to the deposition of lipids and the co-existence of inflammation and impaired inflammation resolution in pan-vasculature, which causes lumen narrowing, hardening, plaque formation, and the manifestation of acute cardiovascular events. Emerging evidence has suggested that vascular circulation can be viewed as a complex homeostatic system analogous to a mini-ecosystem which consists of the vascular microenvironment (niche) and the crosstalk among phenotypically and functionally diverse vascular cell types. Here, we elucidate how cell components in the vascular wall affect vascular homeostasis, structure, function, and atherosclerosis in a holistic perspective. Finally, we discuss the potential role of vascular-stabilizing strategies including pharmacotherapies, natural substances and lifestyle modifications, in preventing cardiovascular diseases by preserving vascular integrity and homeostasis.

## Atherosclerosis and cardiovascular risk factors

**Figure 1:**
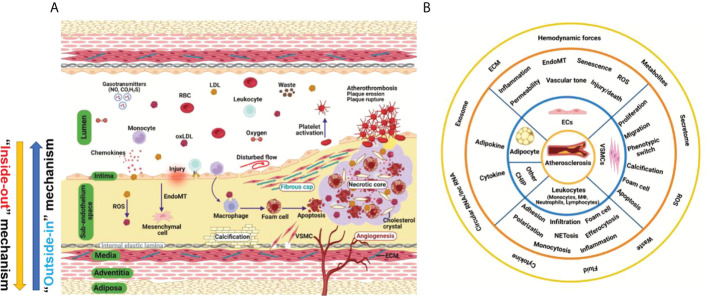
Hallmark of atherosclerosis. **(A)** The pathogenesis of atherosclerosis. Vascular components of atherosclerotic artery which include tunica adiposa, tunica adventitia, tunica media, sub-endothelium space and tunica intima. For simplicity, external elastic lamina in the blood vessel was not depicted. The interplay of different vascular cell types within the local vascular niche drives atherosclerosis development. Both “inside-out” and “outside-in” mechanism contribute to the development of atherosclerosis. **(B)** A holistic overview of hallmarks of atherosclerosis and crosstalk among vascular cell components. Atherosclerosis is complex disease involving different cell types at different vascular layers. The hallmark events in different cell types act in concert to promote atheroprogression. CHIP, clonal hematopoiesis of indeterminate potential; ECs, endothelial cells; ECM, extracellular matrix; EndoMT, endothelial-mesenchymal transition; ROS, reactive oxygen species; VSMC, vascular smooth muscle cells.

Atherosclerosis is a complex and progressive disease with the interplay of multiple cell types and mechanisms involving genetic, epigenetic, environmental, metabolic, clonal hematopoiesis, and lifestyle factors and an evolving landscape (1) (**Figure 1A**). Pan-vascular atherosclerosis is the major cause of cardiovascular disease (CVD) and ischemic stroke (1–3). Atherosclerosis preferentially develops in medium- and large-sized arteries. The blood vessel wall normally consists of tunica intima, tunica media, tunica adventitia and tunica adiposa (4). The blood vessel microenvironment resembles a mini-ecosystem which consists of different cell types, including cells in the blood (red blood cells, leukocytes, monocytes etc.) and vascular cells [endothelial cells (ECs), vascular smooth muscle cells (VSMCs), macrophages and T-lymphocytes etc.)]. These cell types crosstalk with each other *via* ligand-receptor interaction, exosome, endothelial cell secretome as well as cell-matrix interactions, thereby regulating vascular tone and stabilize tissue homeostasis (**Figure 1B**).

Recognized traditional and non-traditional risk factors for CVD include: hyperlipidemia, hypertension, hyperglycemia, hyperuricemia, infection, smoking, male gender, high triglyceride-rich lipoproteins, lipoprotein (a), sleep deprivation, physical inactivity, gut microbiome imbalance, environmental pollution, noise, and psychological stress (1). Taking exercise for example, a recent study (5) has demonstrated that moderate exercise promotes cardiovascular health; however, long-term strenuous exercise damages tunica media, increases aortic stiffness, causes elastic lamina to rupture and media layer thickening of intramyocardial arteries. The authors also found that deleterious remodeling rendered by intensive exercise persisted after withdrawal from exercise (5). Advanced understanding of the novel mechanistic basis of atherosclerosis by addressing how these risk factors causes atherosclerosis will open new avenues to therapeutic interventions aiming to prevent and treat atherosclerosis. In the following section, we summarize the role of cell components in the vascular wall in vascular microenvironment homeostasis and atherosclerosis, with an aim to providing a unified and holistic overview of future research directions in this area.

## Vascular wall components in vascular homeostasis and atherosclerosis

### Endothelial cells in the tunica intima as sentinels in vascular health and pivotal regulator of atherosclerosis

ECs are the key cell type that connects circulating blood and the vascular wall. ECs serve as a natural barrier in the pan-vascular system (6). ECs sense the mechanosignals *via* mechanosensing complex or mechanosensors like plexin D1 (7). ECs serve as the sentinel as well as safeguard for vascular health and tissue homeostasis. The vascular endothelium is thus deemed as a protype of homeostatic regulation of endothelial function (8). Following insults, ECs initiate powerful backup systems/mechanisms to prevent vascular injury. As long as vascular injuries are not overwhelming and reparable, existing cellular defense machineries will be working to maintain vascular homeostasis (8). However, when the injury persists and ECs become activated, leading to endothelial dysfunction, such as inflammation, hyperpermeability, leukocyte adhesion, and cell senescence (6, 9). The earlier stage of low-degree of EC activation is a compensatory tissue repair mechanism that promotes endothelial rehabilitation and preserves vascular health. However, persistent activation of ECs prepares ECs to transit into a decompensatory stage, which leads to endothelial dysfunction (6). After the initial stage of endothelial dysfunction, ECs will recruit more leukocytes to the site of injury, followed by cell adhesion, rolling, crawling, and diapedesis that contributes to atherosclerosis development (6). It is proposed that the protective functions rendered by ECs at resting states include the maintenance of EC quiescence, anti-inflammation, anti-oxidation and anti-proliferation (10).

Beyond canonical physiological functions, ECs also emerge as a new type of innate immune cells (11) which execute a plethora of immune functions, such as cytokine production, phagocytosis of cell debris and bacteria, sensing danger and pathogen-associated molecular patterns, antigen presentation, immune metabolism and trained immunity (11, 12). For example, trained immunity is an immunological concept which refers to long-lasting pro-inflammatory phenotype (similar as innate immune memory) after prior stimulation with or exposure to microbial products and pro-atherogenic stimuli (such as β-glucan, aldosterone, oxidized LDL and LPS), leading to sustained hyperactivation of innate immune system (13). Trained immunity is an important biological mechanism which contribute to atherosclerotic cardiovascular disease *via* epigenetic modification and reprogramming immune-metabolism (13, 14). Recent studies have revealed that, upon infection with virus and bacteria, the anti-viral signaling pathway is activated, the RIG-I (retinoic acid-inducible gene I) pathway in particular (12). Infection with coronavirus, such as SARS-CoV-2, can cause trained immunity in ECs, potentially leading to enhance inflammation in ECs (15).

ECs in their native state also maintain anti-thrombotic and hemostatic functions, which prevent platelet activation and adhesion to activated endothelium. In addition, ECs have active secretory pathways, vesicles and granules to produce huge proteomes to maintain vascular homeostasis and integrity (16). Secreted proteins from ECs play an important role in regulating vascular ECs crosstalk with other vascular cell types. The local microenvironment (niche) in vascular wall and cardiovascular disease conditions can modify endothelial function by regulating ECs’ secretome. Recently, quantitative proteomic analysis of the endothelial secretome (17) has greatly accelerated the discovery of novel secreted proteins from ECs which regulate vascular homeostasis and atherosclerosis. In addition, EC secretome also provides promising biomarkers which have diagnostic and prognostic value for various vascular disorders (16).

More importantly, ECs are the innermost layer of cells that are directly exposed to a wide variety of cardiovascular risk factors, including hyperglycemia, hyperlipidemia, hyperuricemia, hyperhomocysteinemia, and cigarette smoking (12). In addition, a recent study has demonstrated an intimate relationship between cholesterol homeostasis and inflammation/immunity in ECs by showing that pro-inflammatory cytokines upregulated SREBP2 (sterol regulatory element binding protein 2) cleavage/activation and augmented the expression of genes involved in cholesterol biosynthesis *via* NF-kB (nuclear factor-kappa B) and SREBP2 pathway (18).

Other biological functions of ECs include the secretion of nitric oxide (NO) and other vasoactive molecules, such as hydrogen sulfide (H_2_S), prostacyclin (PGI2), endothelin-1 (ET-1) and endothelium-derived hyperpolarization factor (EDHF) (6). By balanced production of these molecules, ECs regulate vascular tone. Furthermore, ECs are highly heterogenous and plastic cell types that can acquire a mesenchymal-like phenotype through endothelial–mesenchymal transition (EndoMT) process *via* the TGFβ/Smads, YAP/TAZ, Snail, Twist, and ZEB family of transcription factors. Undergoing EndoMT, EC-specific markers are lost, and mesenchymal cell-specific markers are acquired, thus impairing normal EC function. EndoMT is strongly implicated in the development of atherosclerotic plaques in mice and in huma patients (6). Endothelial homeostasis is regulated by several master transcription factors, such as KLF2, KLF4 and Nrf2, the activation of which leads to anti-inflammatory, anti-oxidant, anti-thrombotic and inflammation-resolution effects (10, 19). Also, upon injurious stimuli exposure (such as aging, irradiation, anti-cancer therapy, inflammation, virus/bacterial infection and cigarette smoking etc.), ECs will become senescent, and senescence-associated secretory phenotype (SASP) will be acquired, leading to inflammation and dysfunction of neighboring cells (6). The concerted actions of multiple cardiovascular risk factors will cause endothelial cell death and cell loss, leading to plaque erosion and subsequent formation of plaque rupture. Altogether, ECs function far beyond a physical barrier and participate in vital processes in atherosclerosis (20).

### Sub-endothelium space: The fertile “soil” of atherosclerosis

Atherosclerosis preferentially develops in the subendothelial space (SES) of large- and medium-sized arteries in aortic regions with oscillatory blood flow. The SES is a fertile “soil” (microenvironment or niche) for atherosclerosis development due to the intricate interplay among subendothelial low-density lipoprotein (LDL) retention, LDL transcytosis, VSMC proliferation and migration, endothelial dysfunction, foam cell formation, and necrotic core formation (21). After endothelial injury, VSMCs migrate from the media layer to SES and secrete pro-fibrotic extracellular cellular matrix proteins, such as collagens, and fibronectins, leading to vascular remodeling and intimal thickening (21). LDL can pass injured endothelium *via* passive transport or receptor-mediated transcytosis across vascular endothelium. Afterward, LDL can be retained in the SES or oxidatively modified to be oxLDL, which was subsequently uptaken by VSMCs or macrophages to form foam cells, the hallmark of atherosclerosis (22). A proliferation-inducing ligand (APRIL) is a cytokine that binds to heparan-sulfate proteoglycans (HSPGs). APRIL exerts atheroprotective effects by binding to heparan sulfate chains of HSPG2, thus limiting LDL-C retention in the vessel wall, reducing macrophages content and size of necrotic cores (23). Infiltrated monocytes also differentiate into macrophages, which is a classical innate immune cell type that mediates inflammatory response in SES. A recent study (24) has shown that the olfactory receptor *Olfr2 (*human ortholog olfactory receptor 6A2, *OR6A2)* can detect octanal in the circulating blood, leading to interleukin-1β (IL-1β) in macrophages by activating NLRP3 inflammasome. In addition to mediating the activation of inflammasome and inflammation (25), macrophages can also become senescent, and senescent macrophages can accumulate in SES and drive the expression of pro-atherogenic and pro-inflammatory cytokines/chemokines, thus favoring features of plaque vulnerability, including the thinning of fibrous cap and fragmentation of elastic fibers. In contrast, selective clearance of these senescent cells by senolytics prevents atheroma formation (26). Other mechanisms include lipid toxicity induced by free cholesterol and cholesterol crystal, continued buildup of lipid, impaired inflammation resolution, and infiltration of immune cells such as CD3^+^ and CD4^+^ T lymphocytes, fan the flame of inflammation, cell death, and atherosclerosis (15), leading to plaque necrosis and vulnerability. Within the plaques, macrophages are the main decomposing machinery in the atherosclerotic plaque that clears necrotic cell debris *via* the efferocytosis mechanism (27). Other decomposing machinery include autophagy as well as chaperone-mediated autophagy (CMA). Recent evidence has suggested that defective CMA *via* LAMP2A (lysosome-associated membrane protein type 2A) occurs in mouse and human vasculature and that decline of CMA in VSMCs and macrophages promote NLRP3 inflammasome activation, metabolic dysfunction and atherogenesis (28–30).

### VSMCs in tunica media as a key mediator of atherosclerosis

The tunica media consist of multiple layers of VSMCs and is interval-arranged with the elastin lamina. VSMCs are the main scaffold cell type that constitutes tunica media, which is essential for the optimal functioning of arteries by eliciting vasodilation (31). The proliferation and migration of VSMCs are strongly impacted by interaction of VSMCs with extracellular matrix (ECM) proteins, such as proteoglycans, fibronectin, collagen and elastic fibers (32). ECM dysregulation is an important mechanism for disrupted vascular function, atherosclerosis and aortic dissection (32). VSMCs are highly plastic with at least two phenotypes, contractile and synthetic VSMCs, which play an essential role in the embryonic development of vasculature and onset of various diseases (31). Under basal conditions, VSMCs are in the quiescent stage, which is less proliferative and has a relatively low turnover rate. Upon vascular injury, such as chronic hypercholesterolemia or endothelial denudation by drug-eluting stents, the contractile VSMCs switch to synthetic phenotype and undergo proliferation, as well as migration from vascular media to the injury site, to propagate wound repairing (31). During this process, the synthetic VSMCs secrete abundant cytokines and extracellular matrix proteins to create a favorable microenvironment for cell migration and vascular remodeling. During this phenotypic switch, the expression of multiple VSMCs-specific markers is decreased, such as α-smooth muscle actin (SMA), smooth muscle 22 α (SM22α), calponin 1 (CNN1), smooth muscle myosin heavy chain (SM-MHC) and transform to the synthetic phenotype, which contributes to neointima hyperplasia, atherosclerosis and aortic aneurysm (31). In addition, the expression of contractile marker SM22α blocks VSMC-derived foam cell formation *via* augmenting liver X receptor (LXR)-dependent cholesterol efflux (33).

The expression of genes associated with the contractile phenotype of VSMCs is regulated by multiple transcription factors, epigenetic regulators, and noncoding RNAs, such as Myocardin/SRF (34), TET2 (35), SENCR (36), MYOSLID (37), and CARMN (38). While the synthetic phenotype of VSMCs is transcriptionally and epigenetically regulated by krüppel-like factor 4 (KLF4) (35) and NEAT1 (39). In addition to this phenotypic switch, VSMCs can also undergo calcification by augmented expression of calcification-related proteins, such as osteopontin (OPN, also known as SPP1), bone morphogenetic proteins (BMPs), alkaline phosphatase (ALP), the expression of which are regulated by ERK1/2, Runt-related transcription factor 2 (RUNX2) and Wnt signaling pathway (20). Besides, VSMCs can also form foam-like cells after loading with cholesterol, and this source of foam cells from VSMCs has long been underestimated for their role in atherosclerosis (40). Specifically, by genetic inducible fate mapping in mice, medial VSMCs can lose classical VSMC marker genes and transdifferentiate into macrophage-like cells and mesenchymal stem cells in a KLF4 -dependent manner, suggesting high VSMC plasticity in the development of atherosclerosis (41, 42). Single-cell RNA-sequencing studies have confirmed that VSMCs are highly heterogenous, possessing different phenotypes, including senescent, foam cell-like, and osteoblast-like phenotypes (20). Future studies should be directed to elucidate the interaction between VSMCs and vascular niche and how this interaction instructs atherogenesis (20).

The tunica media could be one of the most critical layers in atherosclerosis development. On one hand, being localized underneath the tunica intima, VSMCs are shielded from direct contact with the bloodstream unless the endothelial monolayer is compromised (43). The integrity of the tunica intima fundamentally influences the behavior of VSMCs. Once injured, monocytes could infiltrate into the tunica media and transform into macrophages, which release multiple cytokines and/or growth factors that re-shape the VSMC phenotype (43). Moreover, the platelets could be activated upon vascular injury and release a large number of inflammatory cytokines and chemokines, which induces the phenotypic switch of VSMC from contractile to synthetic phenotype (31). On the other hand, interventions in adventitia also significantly impact tunica media. For example, direct elastase immersion on the adventitia of the aorta could eventually result in the formation of the aortic aneurysm (44). Therefore, it is important to protect tunica media from being disrupted by the external factors (including dietary, environmental factors) from either inner or outer layers of the blood vessel. It also suggests that the progression of atherosclerosis, could be very difficult to alleviate unless VSMC-derived factors are properly controlled.

### Tunica adventitia and tunica adiposa: The “wonderland” of atherosclerosis

The tunica adventitia consists of a more complex mixture of multiple cell types, including fibroblasts, pericytes, macrophages, T cells, dendritic cells, and mast cells etc (4). Most atherosclerosis research has been focused on studying the role of ECs (intimal cells), macrophages/immune cells (SES in disease vessel), VSMCs (media layer cells) in atherosclerosis (20); however, the role of adventitial components and derived factors in atherosclerosis has long been understudied. It is assumed that the cells in the adventitia offer supporting functions to the blood vessel and can also regulate the structure and function of other vascular cells by cell-cell communications, exosome, secretome (such as growth factors, angiocrine factors, cytokines/chemokines, and vasoactive peptides/hormones) etc. (20). Adventitial cells are involved in regulating vascular tone and blood pressure as well as atherosclerosis *via* paracrine and endocrine signaling (45). As plaques in the SES are devoid of innervation. A recent study has elegantly shown that the peripheral nervous system utilizes the adventitia as the main conduit to directly interact with diseased arteries to regulate atherosclerosis *via* the existence of neuroimmune cardiovascular interfaces (NICIs) in adventitia segments (46).

Also, another intriguing and largely unexplored research question is the precise role of perivascular adipose tissue (PVAT) in vascular health and diseases, such as atherosclerosis (45, 47). Recent evidence has implicated PVAT as the fourth layer of the blood vessel wall (the tunica adiposa), which produces adipokines and vasoactive substances (such as leptin, adiponectin, resistin, and visfatin)/cytokines/growth factors that regulate vascular tone/homeostasis (4, 48). One study from Tang laboratory have revealed that periaortic knockdown of ribosomal protein S3A (RPS3A) in mouse PVAT impaired PVAT browning, promoted vascular inflammation and atherosclerosis development by modulating UCP-1 expression in ApoE^-/-^ mice (49). Similarly, another recent study from the Tang laboratory demonstrated that BMP4 (bone morphogenetic protein 4) depletion in PVAT increased endothelial inflammation in an EC/adipocyte co-culture system *via* amplifying IL-1β-driven inflammatory response. More importantly, BMP4 deficiency in adipose tissues exacerbates atherosclerosis, while BMP4 overexpression in adipose tissues promotes PVAT browning and atheroprevention in ApoE^-/-^ mice (50). These findings uncover the important role of PVAT in regulating endothelial function, inflammation and atherosclerosis. Future studies including PVAT in measuring aortic wall’s mechanical behavior, such as aortic stiffness and vasorelaxation/constriction is important (48).

The emergence of single-cell RNA-sequencing and the generation of PVAT-specific *Cre* to precisely manipulate PVAT-derived factors will be useful research tools to answer this question due to the fact that tunica adiposa is a mixture of heterogenous adipocytes (including white, brown and beige adipocytes) (4). The endocrine and paracrine functions of PVAT-derived factors in “outside-in” mechanism of atherosclerosis is an important research direction (4).

## Strategies that stabilize the vascular system to prevent atherosclerosis

**Figure 2:**
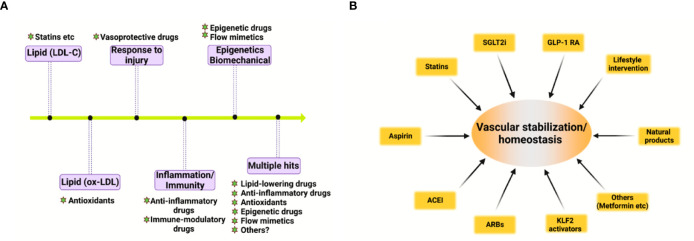
Evolving hypothesis of atherosclerosis and vascular homeostasis-targeted therapies. **(A)** Evolving hypothesis of atherosclerosis. **(B)** Pharmacological and non-pharmacological strategies to promote vascular stabilization and homeostasis and to prevent atherosclerosis. ACEI, angiotensin-converting-enzyme inhibitors; ARB, angiotensin II receptor blockers; SGLT2i, sodium-glucose cotransporter 2 inhibitor; GLP-1 RA, glucagon-like peptide 1 receptor agonist; KLF2, kruppel-like factor 2.

Based on the pivotal role of deregulated vascular homeostasis in atherosclerosis, the “multiple hits” hypothesis of atherosclerosis is gaining more evidence (**Figure 2A**). To this end, several categories of drugs are able to stabilize the “vascular niche” (51) and prevent atherosclerosis (**Figure 2B**). These drugs include statins (lipid-lowering drugs) (52), Angptl3 inhibitors (53), ACLY inhibitors (such as Bempedoic acid) (54), gliflozins (SGLT2 inhibitors, anti-diabetic drugs) (55), glutides (GLP-1 receptor agonists, anti-diabetic drugs) (56), metformin (anti-diabetic drugs) (57–59), aspirin (COX inhibitor, NSAIDs), Angiotensin II converting enzyme inhibitors (ACEI, anti-hypertensive drugs) (60), Angiotensin II receptor blockers (ARBs, anti-hypertensive drugs) (60, 61), naturally-occurring NLRP3 inflammasome inhibitor (colchicine) (62), KLF2 activators (63, 64), AMPK activators (endothelial protective drugs) (65) and many others. Based on the complex nature of atherosclerosis, polypill or ploypharmacology targeting established mechanisms/risk factors are needed. Vasoprotective effects of these drugs include maintenance of vascular homeostasis by generating NO, maintaining normal vascular tone, reducing oxidative stress, inhibiting NF-κB-dependent pro-inflammatory response, and reducing the production of vasoconstrictive molecules (such as AngII and ET-1) as well as stabilizing glycocalyx microstructure in vascular endothelium (8). These strategies also reduce foam cell formation in macrophages by limiting lipid uptake and augmenting cholesterol efflux, attenuating macrophage inflammation and skewing from M1 pro-inflammatory phenotype toward tissue-repairing M2 phenotype (66). In addition, these strategies also halt VSMC proliferation, migration and phenotypic switch from contractile to synthetic phenotype (43). Detailed studies in assessing the effects and mechanism of action of these pharmacotherapies in cell-cell crosstalk warrants further studies.

In addition to these drugs, lifestyle modifications, such as exercise, healthy habitual eating, smoking cessation also maintains vascular homeostasis and prevent atherosclerotic cardiovascular diseases (67, 68). Therefore, pharmacological and non-pharmacological strategies aimed at recuperating vascular stabilization and homeostasis hold promise for anti-atherosclerotic therapies (8).

## Concluding remarks and future perspectives

Atherosclerosis is a progressive disease with a changing landscape which needs to be understood in a holistic view (1). The main theories of atherosclerosis have changed considerably during the past decades. It is increasingly recognized that lipid (both LDL-cholesterol and triglyceride-rich lipoproteins) and inflammation are two predominant mechanisms of atherosclerosis (1). However, the development of atherosclerosis is a slow but progressive process with the interplay of multiple mechanisms. Therefore, a “multiple-hit” hypothesis is possible, including mechanisms of an aberrant lipid profile, inflammation, vascular injury, oxidative stress, hemodynamic forces, epigenetics and others (**Figure 2A**). In this hypothesis, different vascular cell types interact with each other *via* ligand-receptor pairs or proteins in the secretome or cargos (such as miRNAs, circular RNAs, and lncRNAs) carried by exosomes (**Figures 1A, B**). The “multiple-hit” hypothesis of atherosclerosis expands previous established “response-to-injury”, “inflammation”, “LDL oxidation” hypothesis (1), and involve both the “inside-out” (intima-subendothelium-media-adventitia-adiposa) and “outside-in” (adiposa-adventitia-media-subendothelium-intima) mechanisms (**Figure 1A**). This bidirectional mechanism of atherosclerosis reminds us the need to consider cell-cell/cell-environment interaction in atherosclerosis research.

In this article, we reviewed the biological functions of the structural components of the vessel wall in the vascular homeostasis and atherosclerosis. Considering that atherosclerosis is a focal disease that preferentially develops in regions where disturbed blood flow occurs, and the flow pattern is turbulent/oscillatory flow (10, 19), the spatio-temporal and biomechanical basis of atherosclerosis remain to be validated (69). The focal nature of atherogenesis resembles the zonation phenomenon in the liver, which is coordinated by multiple cell types (70, 71). We term this phenomenon “aorta zonation”. Tentatively, the aorta can be categorized into 5 zones, zone 1 is the inner curvature of the aortic arch; zone 2 is the greater curvature of the aortic arch; zone 3 is the arterial branches of the aortic arch; zone 4 is the thoracic aorta; zone 5 is the rest of arterial branches in the aorta. Zone 1, 3, and 5 are predilection sites of atherosclerosis development; however, zone 2 and 4 are protected against atherosclerosis. It is speculated that vascular cells in zone 1 to zone 5 are highly heterogenous and plastic, with different genetic, epigenetic, and metabolic traits. Systems biology techniques such as next-generation single-cell spatial transcriptomics will be an important tool to delineate zone-specific gene expression programs in the aorta and further understand the mechanistic basis of the heterogeneity of vascular cells in different aortic zones and reveal novel therapeutic targets for atherosclerosis. Last but not least, it is of vital importance to elucidate how the vascular microenvironment/niche instructs vascular health and diseases and how homeostatic control of vascular function is achieved. It is well established that atherosclerosis arises and progresses when the homeostatic control is perturbed-pro-atherogenic signaling overwhelms anti-atherosclerotic signaling, leading to complex regulatory loops signaling and atheroprogression (69). Basic and translational research into the mechanisms of maintaining vascular homeostasis raises the exciting prospect of alleviating the ever-growing burden of atherosclerosis by addressing residual cholesterol and inflammation risk in patients with CVD.

## Author contributions

SX and JW conceptualized the review. SX and QL wrote the review. II made figures and edits the manuscript. X-YT provides intellectual input and edits on the manuscript. All authors contributed to the article and approved the submitted version.

## Funding

This study was supported by grants from National Key R&D Program of China (No. 2021YFC2500500), National Natural Science Foundation of China (Grant Nos. 82070464, 81941022, 81530025) and Strategic Priority Research Program of Chinese Academy of Sciences (Grant No. XDB38010100). This work was also supported by Program for Innovative Research Team of The First Affiliated Hospital of USTC (CXGG02), Anhui Provincial Key Research and Development Program (Grant No. 202104j07020051) and Anhui Province Science Fund for Distinguished Young Scholars (Grant No. 2208085J08). Figures were drawn with Biorender.com.

## Conflict of interest

The authors declare that the research was conducted in the absence of any commercial or financial relationships that could be construed as a potential conflict of interest.

## Publisher’s note

All claims expressed in this article are solely those of the authors and do not necessarily represent those of their affiliated organizations, or those of the publisher, the editors and the reviewers. Any product that may be evaluated in this article, or claim that may be made by its manufacturer, is not guaranteed or endorsed by the publisher.
